# Advances in Designing Essential Oil Nanoformulations: An Integrative Approach to Mathematical Modeling with Potential Application in Food Preservation

**DOI:** 10.3390/foods12214017

**Published:** 2023-11-03

**Authors:** Monisha Soni, Arati Yadav, Akash Maurya, Somenath Das, Nawal Kishore Dubey, Abhishek Kumar Dwivedy

**Affiliations:** Laboratory of Herbal Pesticides, Centre of Advanced Study (CAS) in Botany, Banaras Hindu University, Varanasi 221005, India; monishasoni1@gmail.com (M.S.); artiy82440@gmail.com (A.Y.); bhuaks20@gmail.com (A.M.); sndbhu@gmail.com (S.D.); nkdubeybhu@gmail.com (N.K.D.)

**Keywords:** essential oil, modeling-based encapsulation, food preservation, artificial intelligence

## Abstract

Preservation of foods, along with health and safety issues, is a growing concern in the current generation. Essential oils have emerged as a natural means for the long-term protection of foods along with the maintenance of their qualities. Direct applications of essential oils have posed various constraints to the food system and also have limitations in application; hence, encapsulation of essential oils into biopolymers has been recognized as a cutting-edge technology to overcome these challenges. This article presents and evaluates the strategies for the development of encapsulated essential oils on the basis of fascination with the modeling and shuffling of various biopolymers, surfactants, and co-surfactants, along with the utilization of different fabrication processes. Artificial intelligence and machine learning have enabled the preparation of different nanoemulsion formulations, synthesis strategies, stability, and release kinetics of essential oils or their bioactive components from nanoemulsions with improved efficacy in food systems. Different mathematical models for the stability and delivery kinetics of essential oils in food systems have also been discussed. The article also explains the advanced application of modeling-based encapsulation strategies on the preservation of a variety of food commodities with their intended implication in food and agricultural industries.

## 1. Introduction

Essential oils (EOs) have emerged as a fascinating realm of natural bioactive compounds, generating enormous interest in a wide range of sectors such as food preservation, cosmetics, pharmaceuticals, and aromatherapy [1,2]. These bioactive compounds and aromatic liquids are derived from various plant sources, encompassing a wide range of chemical constituents that bestow therapeutic properties with distinctive fragrances [3]. The antibacterial, antifungal, and antioxidant potential of EOs has positioned them as significant alternatives to synthetic additives in food preservation, aligning well with the global shift towards eco-friendly, healthier, and more sustainable consumption patterns [4]. However, the inherent volatility and sensitivity of EOs to environmental factors have posed serious challenges in their direct incorporation into food products. To overcome these limitations, the encapsulation technique has emerged as a promising solution, offering improved stability, controlled release, and enhanced bioavailability of EOs [5]. Encapsulation involves the encompassment of volatile bioactive components within a protective matrix on the basis of computational modeling of different components and material science, thus shielding them from volatilization and degradation while preserving their functionality [6]. 

The diverse array of encapsulation techniques led to the development of encapsulants with superior barrier properties, controlled release mechanisms, and modified compatibility with different food products [7]. The precise manipulation of encapsulation parameters driven by computational insights has enabled the customization of encapsulation matrices for targeted delivery of EOs, ensuring their maximal efficacy as natural green preservatives [8,9].

Computational modeling plays a pivotal role in predicting EO encapsulation and interaction to streamline formulation along with encapsulation dynamics, which accelerates the design of encapsulation systems with specific EO properties and targeted release.

Essential oil emulsions are formulations that combine EOs with water or other aqueous solutions. In addition, the formation of stable emulsions is essential to ensure that EOs are evenly distributed in the aqueous phase and do not separate over time [9]. There are several methods to prepare emulsions of EOs, such as the high-energy mixing method, low-energy mixing method, microfluidization, phase inversion temperature method, surfactant-assisted emulsification, and high-pressure homogenization [10,11]. Further, it is crucial to select an appropriate emulsifying agent, adjust the formulation ratios, and use suitable mixing techniques to achieve stable and long-lasting emulsions [9]. Moreover, the stability assessments of encapsulated EOs focus on their longevity during storage and subsequent incorporation into food matrices. The ultimate measure of success lies in the effectiveness of encapsulated EOs as natural preservatives [12]. Controlled release mechanisms allow EOs to exert their antimicrobial and antioxidant properties over extended periods, contributing to the inhibition of microbial growth and maintenance of sensory attributes in diverse food products [13].

In this comprehensive review, we navigate the multifaceted landscape of EO encapsulation, exploring each facet of formulation, optimization, characterization, stability enhancement, and overall preservation effectiveness with special reference to modeling-based strategies and experimental validation. As the demand for clean-label and natural food preservation solutions continues to escalate, this review also investigates the relationship between modeling strategies and artificial intelligence, emphasizing how these two domains are transforming the vast field of EO encapsulation and, as a result, revolutionizing food preservation industries. 

## 2. Formulation Parameters

### 2.1. Encapsulating Agents/Coating Materials

Coating material can be defined as a membrane or shell that separates the internal phase from its external environment [14]. Micro and nanoformulations of essential oils largely depend upon the choice of coating materials or encapsulants. An encapsulant should possess the following characteristics: food-grade, biodegradable, consideration under the generally recognized as safe (GRAS) category, offer significant safety against oxidation and vaporization of essential oils, compatible with the components of food matrix, large payload, promote sustained release of essential oils, and easy to handle during the manufacturing process and during storage [15,16]. In addition to all these characteristics, cost constraints are one of the major limiting factors for the appropriate selection of coating materials [17].

#### 2.1.1. Polysaccharide-Based Encapsulants

Among all types of delivery systems available, polysaccharides are the most preferred and popular carriers for a large number of bioactive compounds in the food industry [17]. They are commonly used over proteins and lipid-based carriers in terms of their unique properties, such as bioavailability, thermostablility, cost-effectiveness, biodegradability, and ability to interact with both hydrophobic and hydrophilic compounds [18]. Based on their source, polysaccharide-based encapsulants can be classified into four main groups: plant extracts and exudates (e.g., starch and its derivatives, pectin, guar gum, Arabic gum, and gum karaya), animal derivatives (e.g., chitin and its deacetylated product, and chitosan), marine origin (carrageenan, agar, and alginate), and microbial derivatives (e.g., dextran, xanthan gum, and cyclodextrin) [19]. Chitosan is a water-loving, natural straight-chain amino-sugar obtained by the deacetylation of chitin. It is a cationic polymer with gel and film-forming properties and serves as an excellent non-toxic, bio-compatible, and biodegradable polymer for encapsulating essential oils with wider application in food preservation. Chitosan can be used to produce beads, nanoparticles, microspheres, nanofibers, and hydrogels [20,21]. For example, *Pimpinella anisum* essential oil (PAEO) has been ensheathed by chitosan biopolymer via an ionic gelation approach to obtain nanoparticles with improved antimycotic and antiaflatoxigenic potency against food-borne molds contaminating rice. Nanostructured PAEO was found to exhibit higher antifungal and antiaflatoxigenic efficacy (0.08 µL/mL and 0.07 µL/mL, respectively) in comparison to free PAEO [22]. Moreover, another study reported the effect of oregano essential oil (OEO)-loaded β-cyclodextrin microcapsules synthesized by the inclusion process on the storage life of freshly cut purple yam. Encapsulated OEO was successful in extending the storage life of purple yam, and it might also be used on other fruits as a preservative [23]. A similar study reported the efficacy of microencapsulated citral in Arabic gum and sodium alginate via the spray-drying process. Encapsulated citral exhibited antifungal properties and reduced *Fusarium psuedocircinatum* infection by 56% in bananas stored for 9 days at 25 °C. Therefore, citral encapsulated microcapsules could serve as a green alternative to prevent and control the post-harvest losses of bananas [24].

#### 2.1.2. Protein-Based Encapsulants

In addition to polysaccharides, proteins are used as excellent coating materials based on their physical, chemical, and functional attributes, such as emulsification, gel-forming properties, film formation, ability to hold both water and oil, and higher glass transition temperature, which make the polymers suitable for larger application in food industries [25,26]. Some protein-based encapsulants derived from plants include soy protein, maize protein or zein, barley proteins, wheat proteins, potato proteins, and proteins from leguminous plants [10]. Gelatin, milk protein (whey protein isolates and sodium caseinates), and egg protein (albumin) are common animal-based proteins used as coating materials. Among various proteins, plant-based encapsulants exhibit better hydrophobic properties, lesser toxicity, less expensive, and reduced chances in allerginicity compared to animal proteins [27]. *Mentha spicata* L. essential oil (MEO) and magnesium oxide are encapsulated into nanofiber mats using an electrospinning technique with sodium caseinate-gelatin (SC-GE) as coating material. The nanofiber mats were designed in order to determine their potential application in the preservation of fresh trout fillets in cold storage. Eghbalian et al. reported that a combination of SC-GE along with 0.1% magnesium oxide and MEO (0.5% as well as 1%) containing nanofibres was able to limit the proliferation of *L. monocytogenes* and *S. aureus* in fresh trout fillets while also prolonging their storage period to 13 days [28]. A similar study showed the efficacy of encapsulated cinnamon essential oil (CEO) into zein/ethyl cellulose electrospun nanofibres, which greatly prevented the reduction in weight and preserved the firmness of button mushrooms (*Agaricus bisporus*) as well as improving their quality during the storage period [29]. Whey protein isolate (WPI) is a spheroprotein derived from the dairy industry and is widely utilized as a coating material due to its physical, chemical, and functional attributes. Tavares and Noreña [30] investigated the complex coacervation process of ginger essential oil with gum arabic (GA) and whey protein isolate and demonstrated that WPI/GA in a 3:1 (*w*/*w*) ratio produced the best complex coacervate yield. 

#### 2.1.3. Lipid-Based Encapsulants

Lipid-based encapsulants act as the most efficient delivery systems, having several advantages over polysaccharides and protein-based coating materials, such as superior encapsulating efficiency, reduced toxicity, and improved controlled release [31]. Lipid-based nano delivery systems have the ability to entrap a wide range of bioactives with different solubilities (hydrophilic, hydrophobic, and amphiphilic molecules) and have the ability to safeguard bioactives from oxidative damage, pH, and enzymes, ensuring controlled and sustained release of bioactives from nanoparticles [32,33]. Broadly, lipid-based encapsulants can fall into four categories: namely, nanoliposomes, nanoemulsions, SLNs (solid lipid nanoparticles), and NLCs (nanostructured lipid carriers). Various lipid materials used in food applications include fatty acids, glycerides, and waxes such as carnauba wax, bee wax, candellia wax, paraffin, shellac, and phospholipids [14,34]. Solid lipid nanoparticles containing *Zataria multiflora* essential oil have been fabricated using high-pressure homogenization and ultrasonication techniques using glyceryl monostearate, Poloxamer 188, and Precirol^®^ ATO5 as surfactants. The nanoparticles are often spheres of 100 nm in size, ensuring the controlled release of essential oil with great stability, the protection of EOs from enzymatic degradation, and the improvement of EO efficacy in combating fungal pathogens [35]. Kim et al. developed lemongrass essential oil nanoemulsion encapsulated in carnauba wax (18% *w*/*w*) (0.5 to 4.0%, *w*/*w*) using dynamic high pressure (DHP) at 172 MPa for coating plums [36]. The nanoemulsion coating showed promising effects in improving the safety of plums against *S. typhimurium* and *E. coli* O157:H7 by reducing weight loss, phenolic content, ethylene productivity, and respiration rates. Hence, nanoemulsion could be employed to increase the shelf life of plums and prevent them from microbial spoilage.

### 2.2. Surfactants

Surfactants are surface-active agents that are amphoteric in nature, containing a hydrophilic and hydrophobic moiety in their structure. A portion of surfactant has an affinity towards nonpolar media like oil, while another portion has an affinity towards polar media, e.g., water [37]. The hydrophilic–lipophilic balance (*HLB*) is an excellent indication that describes the surfactant’s relative affinity for essential oils and the aqueous phase. The calculation of the *HLB* value of any surfactant is essential in terms of yield and product quality. *HLB* values can be determined theoretically as well as experimentally [38,39]. The following formula can be used to obtain approximate values for most fatty acid esters containing polyhydric alcohols.

$$HLB=20\left(1-\frac{S}{A}\right),$$

where *S* is the saponification number of esters, and *A* is the number of acids. Many fatty acid esters, such as those found in tall oil and rosin, beeswax, and lanolin, do not provide reliable data on saponification [40]. Hence, these could be calculated using the following formula.

$$HLB=\frac{E+P}{5},$$

where *E* is the percentage weight of oxyethylene, and *P* is the percentage weight of polyhydric alcohols (such as glycerol and sorbitol). According to Griffin, the above formulas are satisfactory for various non-ionic surfactants [41]. However, non-ionic surfactants containing propylene oxide, butylene oxide, nitrogen, and sulphur exhibited different behavior apart from their composition. Furthermore, the *HLB* values of ionic surfactants cannot be calculated on a weight percentage basis because, even if the hydrophilic portions have low molecular weight, its ionization lends more emphasis to that portion, making the product more hydrophilic. For such products, experimental techniques must be utilized [42].

$$HLB=\sum HLB_{i}\times f_{i},$$

where $f_{i}$ is the weight fraction of surfactant *i*. According to Griffin (1949), the amount of *HLB* required (*HLB_r_*) for the emulsification of blended oils can be calibrated using the following formula [42].

$$HLB_{r\;}=\sum HLB_{r}\times f_{i},$$

where $f_{i}$ is the weight fraction of *i* oil. The *HLB* value is actually an empirical number ranging from 0 to 20. The surfactants with *HLB* values more than 10 have a high affinity for hydrophilic molecules (water), whereas surfactants with *HLB* numbers less than 10 have a high affinity for lipophilic molecules (oil) [43]. A comprehensive study was conducted by Nirmal et al. to determine the optimal *HLB* for the attainment of lemon myrtle (LMEO) and anise myrtle (AMEO) essential oil-based nanoemulsions [44]. Different possible combinations of Tween 80 and Span 80 with *HLB* values of 15 and 4.3, respectively, were prepared. The minimum droplet size and PDI (polydispersity index) were attained with surfactant systems with *HLB* values of 14 and 12 for LMEO and AMEO nanoemulsions, respectively. Moreover, surfactants reduce the interfacial tension, preventing aggregation of droplets during homogenization. Thus, the concentration of surfactants is an important issue under consideration due to their large surface area [37]. 

Another often-employed approach for choosing the optimal ratio in the presence of two surfactants is using the *HLB* method, which yields the mass ratio of each surfactant so as to achieve the required *HLB* [45].

$$M_{a}=\frac{\left(HLB_{d}-HLB_{b}\right)}{\left(HLB_{a}-HLB_{b}\right)},$$

where *M_a_* is the surfactant’s mass ratio, *HLB_d_* is the desired value of *HLB*, and *HLB_a_* and *HLB_b_* are respective *HLB* values of the two surfactants *a* and *b*. 

The selection of surfactants for food application is one of the most crucial factors that can affect the production procedure, nanoemulsion stability, and performance [46]. Surfactants have a significant impact on electrostatic or steric repulsions at the interface, which rely on the structure of the surfactant (like branching, electrical properties, or aromaticity). Added elements, such as pH, temperature, electrolyte type, and the presence of additives, are also important for emulsion preparation. Surfactants can be classified into three types: ionic (includes both cationic and anionic), non-ionic, and amphoteric (zwitterionic) [47]. 

The production and stability of any micro or nanoformulation are greatly affected by the electrical properties of the surfactant being used. This factor has an effect on the stabilizing mechanism of the surfactant’s polar head in an aqueous medium. In fact, electrostatic interactions stabilize ionic surfactants, while dipole interactions, hydrogen bonding, and repulsive forces caused by steric hindrance stabilize non-ionic surfactants [48]. The latter, on the other hand, are the primary choice since, in comparison to ionic surfactants, they have a safer toxicological profile and are widely approved even for oral intake [49]. Some commonly used surfactants include sucrose esters, sorbitan esters (such as Span and Tween series), glycerol fatty acid esters, polysorbates, and polyoxyethylene ethers. The polysorbates Tween 80 and Tween 20 are the two most commonly employed surfactants for the development of essential oil-based colloidal systems since they have been shown to provide stable formulations without the application of a co-surfactant. This is useful from both a formulation and a toxicological standpoint [47,50]. A study has been conducted to formulate trans-cinnamaldehyde (a natural antibacterial compound) nanoemulsion in order to enhance its bioavailability and antibacterial activity. To improve the stability profile, a nanoemulsion containing trans-cinnamaldehyde and 1,8 cineol (as carrier oil) was formulated using an ultrasonication technique with emphasis on three different factors: the type of surfactant used, e.g., whether Tween 80 or Tween 20 used, SOR (surfactant-to-oil ratio), and the time for the sample to be sonicated. The results revealed that small particles were formed (27.7 nm in size) when Tween 80 was used as a surfactant with a surfactant:oil ratio (2:1 *w*/*w*). Sonication for 15 min was determined to be the best fabrication environment with increased stability and powerful antibacterial activity [51].

### 2.3. Co-Surfactants

A co-surfactant is often an amphiphilic surface-active component that cannot stabilize an emulsion on its own due to its small polar head. Instead, it aids in the synthesis of microemulsions (MEs) and nanoemulsions (NEs) by synergistically supporting the surfactant’s function [47]. A co-surfactant can perform various functions in emulsion formation, as follows.

➢ Decreasing the interfacial tension to near zero;
➢ By putting themselves in between the surfactant tails, enhancing the interface’s pliability and fluidity;
➢ Reducing the total viscosity to prevent the production of more stiff structures like gels and liquid crystals;
➢ Frequently demonstrating solubility in both aqueous and organic phases, thus aiding in solubilizing compounds with poor solubility (like vitamins, essential oils, and phytosterols);
➢ Helping to connect the water/oil and oil/water regions via bicontinuous regions, causing a feasible transition from one regime to another to be facilitated without phase partitioning [52,53,54].

Despite the negative free energy associated with the preparation of MEs, the co-surfactants enhance the process of ME preparation. Moreover, the presence of co-surfactants is also essential for fabricating NEs using low-energy approaches [55,56]. Some examples of commonly used co-surfactants include ethanol, glycerol, sodium deoxycholate, caprylic acid, propylene glycol, 2-Pyrrolidone, butylene glycol, and isopropanol. Generally, alcohols with short or medium chains are the most prevalent co-surfactants. The reason they are most suitable is because they are tiny, amphiphilic molecules with a hydrocarbon chain and an OH^−^ group. They quickly diffuse between the lipophilic and aqueous phases, arriving at the junction of two phases. The co-surfactant molecules migrate between the molecules of the surfactants, decreasing interactions between the polar heads and hydrocarbon tails. As a result, the interfacial coating becomes flexible and more prone to deform around droplets [57]. Alcohols may also affect the soluble nature of essential oils and aqueous phases since both phases are immiscible and show significant partitioning. It is interesting to note that some EOs contain monoterpene alcohols, such as geraniol, terpinen-4-ol, and α-terpineol, which act as co-surfactants to promote the formation of micro emulsions and provide the molecules with amphiphilic properties [58,59]. Cassia oil microemulsion was formulated by Xu et al., using cassia oil, ethanol, and Tween 20 in a ratio of 1:3:6 (*w*/*w*/*w*), which inhibited the fungal growth and the microemulsion was effective in controlling the post-harvest infection of citrus fruits by *Geotrichum citri-aurantii* [60]. Similarly, nanoemulgel containing *Piper betle* essential oil was fabricated with carbopol 940 as a gelling agent. The droplet size of the nanoemulsion ranged from 28 to 161 nm. The nanoemulgel was found to be an efficient carrier system for *Piper betle* essential oil with potential transdermal delivery [61].

### 2.4. Oil Phase and Other Components

The heart of any oil-in-water emulsion is the oil phase, which significantly affects the production and stability of MEs and NEs. The features of nanoemulsions like the size of droplets and stability can be changed by factors linked to oil, including polarity, water solubility, interfacial tension, and viscosity [62]. The formation and stability of droplets are greatly influenced by the oil-to-water and oil-to-surfactant ratios [63]. Additionally, droplet stability depends on the extent of the solubility of oil, which, in turn, depends upon the ratio of hydrophilic to hydrophobic oil components since oils can provide a mixture of hydrophilic and hydrophobic molecules. Excessive concentrations of ether-soluble components usually cause Ostwald ripening, which is the primary cause of instability in nanoemulsions [39]. The lipophilic components, especially oils and other bioactive ingredients, can be encapsulated into nanoparticles to improve their physical stability, increase their bioactivity, prevent degradation from environmental conditions, regulate their release, make it easier to manipulate or incorporate them into aqueous formulations, and avoid direct interaction with the food ingredients [64]. This technology has also been utilized extensively in drug delivery systems and allows for the regulated release of medications with biological affinities [65]. When utilized as a preservative in food systems, nanoencapsulation technology also makes it possible to use naturally occurring bioactive substances at lower concentrations, reducing the potential side effects for consumers. Here, our main focus lies on explicitly utilizing essential oils as an oil phase in formulating various MEs and NEs.

Essential oils are natural, volatile, complex molecules with strong aromas produced as secondary metabolites by aromatic plants. Essential oils can be extracted from leaves, stems, flowers, roots, seeds, fruit rinds, bark, resins, etc. [66]. The technique of producing essential oils using steam or hydro-distillation was discovered by Arabs in the Middle ages. Essential oils are used in nature to protect plants against herbivory, acting as pesticides, insecticides, antibacterials, and antiviral agents. They may also attract some insects to aid in pollination and seed dispersal while repelling others [67]. Essential oil extraction is the most time-consuming and labor-intensive process. The most common methods of extraction include maceration, cold pressing, enfleurage, hydrodistillation, solvent extraction, and supercritical CO_2_ extraction [68].

Aromatic oils are extensively used in the preservation of food, embalming, and as antibacterial, analgesic, sedative, anti-inflammatory, and anaesthetic treatments because of their bactericidal, fungicidal, and therapeutic potentialities [69]. Regardless of the preservative efficacy of essential oils in food systems, some limitations in their practical application have been identified because of intense aroma, high reactivity, hydrophobicity, low solubility, and possible detrimental interactions with various components of food, resulting in changes in organoleptic attributes [4]. Several current technology improvements involving various delivery techniques have been used to address these shortcomings. Nanoencapsulation of essential oils is one of the revolutionary and innovative delivery systems that improves antimicrobial efficacy through improved stability, dissolution, and regulated release of essential oil aroma in the food system, as well as protection against environmental factors such as light, O_2_, moisture, and pH [70,71]. A large number of bio-polymeric substances such as chitosan, starch, cyclodextrins, alginate, and many others have been employed as coating materials for EO encapsulation due to their biodegradability, biocompatibility, and eco-friendly properties. Therefore, encapsulated EOs may be the finest non-toxic and sustainable substitutes for synthetic preservatives for their wide application in the agriculture and food industries [47].

Apart from all these components, other components could also be found in microemulsions and nanoemulsions, usually for providing stability. In order to harmonize oil phase density with the water phase, thickening agents are specifically utilized in oil-in-water emulsions. Therefore, they could prevent the onset of creaming or sedimentation events by acting on forces of gravity [47]. Additionally, texture modifiers are frequently employed. Hydrocolloids with thickening abilities can change the system’s rheology and texture, which increases stability by preventing gravitational separation [72]. The role of Wo, i.e., water-to-surfactant ratio, is also a very important parameter. Keeping the surfactant amount constant and changing the amount of water content, there is a significant change in the water pool, e.g., the size of the reverse micelle, which holds the reactant molecules and is the site of reaction. Therefore, a change in Wo directly affects the size of the finished product [54]. Finally, the different components or encapsulants facilitate the intended application of encapsulated species with modified core material and wall matrix properties [73].

## 3. Methods for Preparation of Nanoemulsion Involving Different Components

**Figure 1:**
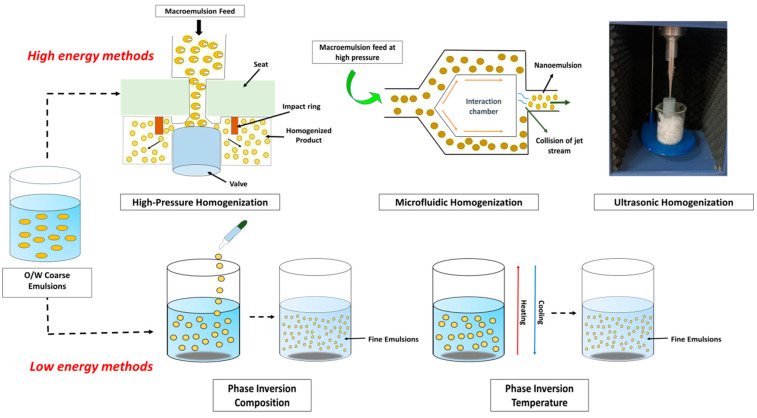
Strategies for encapsulation of essential oil.

Micro/nanoemulsions are fabricated utilizing emulsification techniques that combine high and low-energy inputs [74]. Fabrication methods employed for ME and NE formation differ due to variability in the free energies of the systems. The formation of microemulsions is an energetically favorable process; hence, by combining different components, they can be formed spontaneously. ME formation is fueled by a dynamic process based on the following equation:

ΔG=γΔA−TΔS,

where ΔG is the Gibbs free energy, *γ* is the interfacial tension of O/W emulsion, ΔA is the change in the interfacial area, *T* is the temperature, and ΔS is the change in entropy of the system [47]. In contrast, the production of NEs requires an external source of energy (due to greater surface area) that is greater than ΔG, which is positive in the case of thermodynamically unstable systems. Hence, both high and low-energy methods can be used for NE formation [75]. Although low-energy methods typically produce NEs with smaller droplet sizes, they are constrained in the types of oils and surfactants that may be used and even need larger concentrations of surfactants. High-energy techniques, however, are more adaptable because they can utilize a variety of oils and surfactants [76]. The different high-energy and low-energy methods are discussed below and represented in Figure 1. 

### 3.1. High Energy Methods

Large disruptive forces, like compression, collision, and cavitation, are generated in high-energy methods using mechanical instruments such as sonicators, microfluidizers, and high-pressure homogenizers that create tiny droplets of liquid. The equipment, production conditions, and various other factors, such as the sample’s composition and characterization, as well as time and temperature, are responsible for determining droplet size [77,78]. High-energy techniques demand sophisticated machinery and use a lot of energy; hence, it is an expensive technique. Although these high-energy techniques are effective in reducing particle size, they are not going to work on drugs that are temperature-sensitive, as well as macromolecules like proteins, enzymes, retinoids, peptides, and nucleic acids [79]. This is due to the fact that high-energy technologies operate at high temperatures and pressures, which have the potential to harm proteins and drugs that are thermolabile or sensitive [80]. 

#### 3.1.1. High-Pressure Homogenization (HPH)

High-pressure homogenization is the most widely used technique to produce nanoemulsions. This technique uses a high-pressure homogenizer or a piston homogenizer to create nanoemulsions with small particles (approximately 1 nm in size) [81]. The macroemulsion is forced to travel through a tiny aperture where pressure in the range of 500–5000 psi acts on it, leading to the formation of extremely small-sized droplets as a result of the interaction of many factors, including cavitation, hydraulic shear, and severe turbulence. This method can be repeated until the required droplet size and polydispersity index are achieved [82]. Unless coalescence occurs, the droplet size will continue to decrease as the pressure difference increases. Increasing the pressure differential or the energy density in high-pressure homogenizer will reduce the droplet diameter until coalescence occurs. However, the disruption unit has an effect on droplet sizes because it regulates flow patterns, which, in turn, causes droplet disintegration [79]. Droplets disintegrate when the disruptive energy becomes equal to the viscoelastic energy and surface energy.

Disruptive energy=Viscoelastic energy+Surface energy,

$$Disruptive\;energy=\frac{\pi }{6}d^{3}\tau ,$$

where *d* is the droplet’s diameter, and τ is the extensional energy per unit volume.

τ=ɳ×γ,

where ɳ is the viscosity of the continuous phase, while *γ* is the shear rate [83].

#### 3.1.2. Microfluidic Homogenization (MH)

Nanoemulsions can be fabricated using high-pressure microfluidic devices [84]. In fact, this technology has facilitated the mass manufacture of size-tailored nanoemulsions. Both HPH and MH require high pressure to reduce the droplet size; still, MH is reported to be more effective than HPH since it can generate nanoemulsions of the same size in a single pass at an equal working pressure [78]. In the case of microfluidizers, in the nozzle, there is an interaction chamber where macroemulsions feed jets from two opposite channels where a collision occurs. An air-driven pump is generally utilized, which can compress the air to pressures ranging from 150 to 650 MPa, which provides motility to the macroemulsion feed. Due to high-pressure conditions, the macroemulsion feed stream is forced to go via microchannels, leading to a collision between the two opposing channels, thereby generating a tremendous amount of shear force [85]. As a result, fine emulsions are formed. The Weber number (*We*) of the droplet is a dimensionless number in fluid mechanics, which describes the ratio of the droplet’s kinetic energy due to inertial forces (*E_F_*) affecting the energy due to interfacial tension (*E_T_*).

$$We=\frac{E_{F}}{E_{T}}=\frac{\rho_{C}u^{2}d}{\gamma },$$

where *ρ_c_* is the density of the dispersion medium (continuous phase), *u*^2^ is the mean square spatial change in liquid velocity in distance *d* (droplet size), and *γ* is interfacial tension [86]. It can also be stated that the Weber number corresponds to the ratio between drop disruption and drop-maintaining forces. Drop disruption or drop breakup occurs when the Weber number hits the critical level (*We_critical_*) [87].

$$We>We_{critical};Droplet\;breakdown\;occurs,$$

$$We<We_{critical};Droplet\;deformation\;occurs.$$

Magarvey and Taylor [88] demonstrated the droplet-breakup occurring in bag mode using free-falling water droplets with diameters ranging between 1 cm and 2 cm. The approximate calculated values of the critical Weber number ranged between 3.5 and 14 for these experiments. After evaluating the experiments of Lane et al. [89], Taylor discovered that the average critical weber number for the disintegration of water droplets across a wide range of conditions was 2.7. It can be suggested that the critical Weber number is a function of liquid attributes other than surface tension [90]. Hanson et al. [91] also reported that the value of the critical Weber number ranged between 4.8 and 24 when fluids of variable viscosity were taken into consideration. 

#### 3.1.3. Ultrasonic Homogenization (UH)

The technique of sonication or ultrasonic homogenization can be easily utilized to generate kinetically stable nanoemulsions [85]. In general, a sonicator probe is brought close to the dispersion medium containing a dispersed phase, surfactants, and co-surfactants, resulting in massive mechanical vibrations as well as cavitation, which provides the necessary energy input required for droplet formation at the nanoscale [80]. Ultrasonic waves are produced using a piezotransmitter that converts electric charges into mechanical vibration [92]. The frequency or amplitude of the ultrasonic waves, power, and sonication time are the most crucial parameters determining the nanoemulsion’s morphology (i.e., particle shape and size). 

### 3.2. Low-Energy Methods

Low-energy emulsification methods are becoming increasingly important for micro-/nanoemulsion preparations since high-energy methods require high-energy levels and are expensive [93]. Low-energy emulsification technologies employ the system’s intrinsic chemical energy, i.e., the chemical potential of the components involved require very little energy since only basic stirring is required. They generally allow for the production of small droplet sizes comparable to high-energy droplets [94]. Rapid diffusion of the surfactant causes the production of nanoemulsion with low-energy approaches without introducing the solvent from the discontinuous phase to the dispersion medium. This occurs as a result of spontaneous curvature shifting of the surfactant, generally called “self-emulsification” [75]. This shifts from negative to positive in the case of oil-in-water (O/W) emulsion, while it shifts from positive to negative in the case of water-in-oil (W/O) emulsion [95]. The most frequently utilized low-energy methods are discussed below.

#### 3.2.1. Spontaneous Emulsification (SE)

Spontaneous emulsification refers to the spontaneous formation of emulsions, generally when two immiscible liquids, oil and water, are mixed together at a specific temperature. This approach does not require any extra equipment [96]. It primarily depends upon the following parameters: concentration of surfactant, molecular structure of surfactant, interfacial tension, phase transition area, and interfacial and bulk viscosity [97]. In pharmaceutical industries, these systems are known as self-emulsifying drug-delivery systems (SEDDS) or self-nano-emulsifying drug-delivery systems (SNEDDS) [82]. This approach relies upon the rapid movement of water-dispersable ingredients such as surfactant, co-surfactant, and solvent from an oil into an aqueous phase. The oil–water interfacial area increases as a result of the enormous turbulence caused by the rapid movement of water-miscible components into an aqueous phase at the biphasic interface, which leads to spontaneous emulsification [77].

#### 3.2.2. Emulsion Phase Inversion (EPI)

The concept of EPI is the opposite of SE. In this case, the lipophilic phase is continuously agitated using a stirrer, and the aqueous phase is slowly and continuously added to it. Here, the lipophilic phase is a mixture containing essential oils with various surfactants and co-surfactants. Since the EPI approach also requires mixing water with oil, it is also sometimes called catastrophic phase inversion (CPI) [77,98]. Phase transitions are due to the alterations in the spontaneous curvature of the surfactant and can be produced at a constant temperature by altering the chemical composition of the system using the phase inversion composition (PIC) method or by maintaining constant chemical composition by modifying the temperature using the phase inversion temperature (PIT) method [80]. These technologies have some drawbacks, including complexity, the need for accuracy, and the usage of artificial surfactants.

#### 3.2.3. Phase-Inversion Composition (PIC)

In this method, there is an alteration in the composition of the system while maintaining a constant temperature. Nanoemulsions are prepared by the addition of water or oil in a continuous manner to a mixture containing either oil–surfactant or water–surfactant [95]. Since it is simpler to add a component to an emulsion rather than producing a sudden shift in the temperature, the PIC approach is better suited for the large-scale production of nanoemulsions as compared to the PIT method. The volume fraction of water in the system is increased as more water is added, resulting in a transition composition. In other words, when the surfactant’s polyoxyethylene chains become more hydrated, its spontaneous curvature shifts from negative to zero [80,82]. The bi-continuous phase will start as phase inversion happens. The development of droplets in droplet inclusion is likely caused by the entrapment of one phase into the other, which is encouraged by the intermediate bicontinuous structure [79].

#### 3.2.4. Phase-Inversion Temperature (PIT)

This method involves a change in the temperature of the system while maintaining a constant composition. In the PIT method, the mixing of non-ionic surfactants, water, and oil occurs at room temperature. The emulsification process is accomplished by altering the affinity of surfactants for the aqueous phase and essential oils by providing variability in the temperature [99,100]. As the temperature rises, the mixture exhibits a positive curvature, and the polyethoxylated surfactant becomes hydrophobic (due to dehydration) and soluble in oil [82]. Phase inversion occurs as a result, and the O/W emulsion converts into a W/O emulsion with a negative curvature. It is important to note that as the curvature approaches zero at intermediate temperatures, also known as the *HLB* temperature (when the surfactant’s hydrophilic and lipophilic qualities are balanced), highly unstable emulsions are generated [97]. In order to produce stable nanoemulsions, a quick change in temperature is feasible; a rise or fall in *HLB* temperature by 25–30 °C prevents coalescence of droplets. The major drawback of the PIT method is that they become more susceptible to coalescence when the temperature of the dispersed phase (droplets) rises [80]. In this context, understanding the phase behavior of the surfactant, hydrophilic–lipophilic balance, solvent solubility, viscosity, speed of homogenization, shear force, and temperature could have an important role in the development of stable nanoemulsion with intended application in food-based industries [101].

## 4. Various Strategies for Encapsulation of Essential Oils on a Nanometric Scale

Food contamination by spoilage and pathogenic microbes poses a significant threat to public health, food security, and sustainability. As a result, essential oils have become increasingly popular in the past few years because of their antioxidant potential and antimicrobial characteristics, which protect food from rotting. However, essential oils can be effective antimicrobials, but their chemical and biological liability and hydrophobic nature strongly limit their use as antimicrobial food additions [47]. Encapsulation is an efficient method for overcoming these disadvantages. Various encapsulation technologies are being investigated as a means of stabilizing essential oils, disguising their odors, and possibly boosting their antimicrobial effectiveness through more continuous antimicrobial release [12].

### 4.1. Physical Methods

Physical approaches do not involve the polymerization process since the used material or matrix is a polymer. Thus, only the mechanical formation of the microcapsules occurs [102].

#### 4.1.1. Extrusion as an Encapsulation Method

Extrusion encapsulation is most commonly employed to encapsulate organic substances, which are volatile in nature and unstable like EOs. It is exclusively employed with glassy carbohydrate matrices. This technique is widely implemented in food industries, pharmaceutical sectors, and cosmetics [103]. Extrusion techniques vary depending on the type of extruder, the state of extrusion, and additional factors, such as the kind of extrudate generated. For example, the co-extrusion method has been employed to design microgels encapsulating kenaf seed oil [104]. The coating material used in this study consisted of a mixture of alginate and high-methoxyl pectin alginate, which showed high oil retention efficiency. This combination was fabricated at low shell–low core flow rates in the range of 7.0–0.2 mL/min and, with a vibration frequency value of 500 Hz, was found to be highly efficient in encapsulating maximal kenaf seed oil. There are four types of extrusion technologies:

Hot-melt extrusion is a process that requires a minimal quantity of water. The process is fast, continuous, and requires minimal unit operations. There is high-throughput, excellent material mixing (encapsulant and active component), and restricted oxygen exposure in the channel where extrusion occurs. Bioactives can be injected at different time intervals throughout the extrusion process; their residence time is brief and can be tracked online. Hot-melt extrusion is used to stabilize bioactive chemicals, which improves controlled release, flavor mask ink, and particle size uniformity (500–1000 nm) [105]. 

Melt injection is a type of vertical extrusion technique in which the bioactive ingredients are disseminated in a cold bath containing dehydrating liquids such as liquid N_2_ and isopropanol after being dispersed in a hot bath containing melted polysaccharides. The coating material or the encapsulant solidifies upon coming into contact with cold bath conditions, thus trapping the essential oils or different bioactives inside the encapsulating matrix [106]. It has been predicted that using the melt injection technique retains the essential oils or different bioactives for around 5 years compared to other techniques, particularly spray drying, which reports retention for around one year [107].

Encapsulation of essential oils and pro-biotic bacteria is usually carried out using the co-extrusion technique since it requires less heat compared to the hot-melting extrusion technique. It generally employs alginate as a coating material. Co-extrusion technology has a number of benefits over conventional encapsulation strategies, such as increased retention period of encapsulated bioactives, improved stability, and protection against oxidative damage [108]. One of the major constraints using this approach is unevenness or variability in the composition of wall materials, which refers to the blending of one or more coating agents with diverse compositions and structural variations. Because of this non-uniform nature of wall materials, there is poor encapsulation efficiency and interfacial destabilization of bioactive components [109].

Electrospinning and electro-spraying are single-step, simple processes for synthesizing microcapsules and nanocapsules in dried form. These processes are excellent for encasing heat-labile materials since the process functions at average atmospheric pressure and temperature [110]. When polymers are introduced into a hardening solution, allowing the electrostatic charges to travel through it, this approach is utilized to create polymeric microspheres. This method works on the principle of applying an electric field between a positively charged needle and a collection unit to disturb the liquid strand at the mouth of the needle, creating a charged stream of little drips [111].

**Figure 2:**
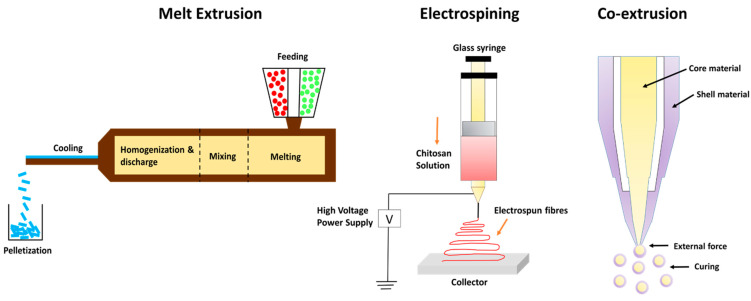
Types of extrusion technology.

Electrostatic extrusion is advantageous in the food business because this technique helps to synthesize polymeric microspheres with reduced particle size [112]. Electrostatic extrusion utilizes less energy, minimizes manufacturing costs, and delivers encased bioactive substances with unique features. Their major drawback is limited throughput. This disadvantage prevents extensive commercial utilization [110]. Figure 2 describes different types of extrusion methods for the encapsulation of EOs.

#### 4.1.2. Fluidized Bed Coating

The fluidized bed coating method is utilized to produce encapsulated particles on a fluidized bed powder by spraying the coating material on it at an optimized rate to allow the coating material to suspend without expelling them from the stream [113]. The efficiency of fluidized bed coating depends upon a variety of processing factors, including the rate of solid circulation, humid conditions, rate of coating feed provided, temperature, and nozzle atomization. All these factors are important because they have an impact on the agglomeration of particles and the development of film [114]. As a result, optimizing coating operations employing a fluidized bed approach is dependent on determining the impact of processing parameters. Encapsulating agents like starch, gums, and proteins provide better protection and stability over longer periods as compared to other coating materials [115]. Maltodextrins have been widely employed as spray-drying wall materials because they are highly soluble in water, less viscous, contain low sugar, are cost-effective, and produce colorless solutions. All these features owe to their extensive application in food industries [116]. The fluidized bed coating method is highly efficient in generating a homogenous coating encircling the particles, strengthening the barrier qualities, and safeguarding bioactive components from damage since the coating material forms a protective sheath around particles [117]. Encapsulation via a fluidized bed is thought to be effective for water-soluble solid particles, particularly in low-pH active substances, in protecting other ingredients in fortified foods [118]. It is a technology that was originally created for the pharmaceutical industry, but it has found widespread use in functional additive formulations in the food sector [119]. Fluidized bed coating has some technological drawbacks and difficulties as this technology employs high temperatures, which may be harmful to some components that are easily oxidized at high temperatures; sometimes, there is a loss of bioactive components when the temperature far exceeds the melting temperature of encapsulating agents, the utilization of organic solvents, and failure to effectively encapsulate submicron-sized particles [118]. 

#### 4.1.3. Spray Drying

Spray drying (SD) is an extensively used industrial process for encapsulating food components [120]. This technology is cost-effective, simple, continuous, and easy to scale up. In SD, an aqueous emulsion is generated using core components and wall materials, which are homogenized, followed by atomization in a drying chamber, ultimately dehydrating the spray-dried particles [121]. The spray drying technique is divided into three steps, namely, producing an active aqueous emulsion containing bioactive components, homogenizing the emulsion, and lastly, passing the final solution into the drying chamber for atomization. On the other hand, higher air input temperatures (150–205 °C), even for just a short time, may cause thermos-sensitive chemicals to deteriorate [122].

The physical and chemical attributes of spray-dried raspberry and vanilla fragrance powder were investigated by Jedlińska et al. [123]; they confirmed that vanilla flavors retained less aroma, but raspberry flavor powder had a small size of particles, reduced flow ability, low bulk density, and a greater glass transition temperature (Tg).

Emulsified essential oils are typically disseminated in a solution including wall materials, which act as initial feed for the spray drier. Spray drying can result in fine powders containing solid particles ranging in microns [124]. Huang et al. confirmed this while studying four different coating agents for encapsulation via the spray-drying approach to boost the stable nature of volatile yogurt-flavored bases, namely, cyclodextrin, octenyl succinate anhydride, chitosan, and maltodextrin [23]. They discovered that, amongst all, chitosan was best in terms of the physical attributes of any coating agent, having better encapsulation efficiency, microstructures with smooth surfaces, and superior water soluble properties and stability. 

#### 4.1.4. Spray Chilling

Spray chilling (spray cooling) is an encapsulating process that involves spraying fluid into a chamber containing cold conditions to enhance the solidification process of particles, resulting in the production of a powder [125]. It entails dispersing the bioactive component into molten wax or fat, which acts as an encapsulant, then homogenizing the dispersion and atomizing it in a cool chamber at a precise temperature determined by the melting temperature of the encapsulant used for the process [10]. Compressed air is employed after the melting of the encapsulant to ensure appropriate dispersion of the bioactive component into the molten encapsulating matrix [126]. This approach differs from spray drying in that particle processing has no drying stage or moisture loss from the capsule surface. Interestingly, Günel et al. found that spray freezing may successfully decrease capsaicin’s pungency while preserving its biological activity [127]. The Scoville heat unit (SHU) of capsaicin powder encased inside wall material was lowered by 4500 times in comparison to pure capsaicin. This study supported commercial spray cooling microencapsulation of capsaicin in order to make it palatable for those who are unable to ingest it due to pungency aversion.

Spray chilling is especially useful for components with a volatile nature, which are prone to thermal destruction, such as EOs. The solid matrix within the particles helps in the retention of EOs because the machinery used here is usually operated at low temperatures; hence, this method saves money on energy [73]. Hydrogenated vegetable oils and wax with high melting temperatures (32–42 °C) are frequently utilized as coating materials for encapsulating water-soluble bioactives, including vitamins, since they have the capacity to control the release patterns of bioactive components in moist conditions [128]. For example, investigations have revealed that essential oils were released from powders generated via the spray-chilling method within a short time period (a few minutes) after integration into food matrices, which signifies that most of the essential oil was present on the surface of particles [129].

#### 4.1.5. Freeze Drying

Freeze drying, or lyophilization, is an effective method for dehydrating colloidal delivery systems that incorporate bioactive compounds sensitive to temperature, such as essential oils. The freeze-drying method usually consists of three steps: (I) creating an emulsion containing bioactive compound and a suitable coating material; (II) allowing the suspension to freeze; (III) removing the aqueous component (especially water) by vacuum sublimation [130]. Powders with complicated characteristics have been created using the freeze-drying technique. When this frozen solution is subjected to extremely low-temperature conditions, the sublimation of the ice crystals occurs. It is an expensive drying process because it requires the use of a vacuum pump, a large amount of energy expenditure, and 24 to 48 h of incubation time [131]. Ocampo-salinas et al. [132] performed encapsulation of condensed vanilla extract using a freeze-drying technique followed by micro-fluidization, employing maltodextrins as wall materials. The research demonstrated the formation of vanilla extract microcapsules via a freeze-drying approach with more than 95% encapsulation efficiency. Freeze-drying was used to successfully encapsulate limonin utilizing β-CD as wall material with core-to-coating efficiency ratios of 68.14% and 80.52% at 1:10 and 1:20, respectively. 

This procedure is easy to operate and yields superior-quality powders but is impractical for many commercial uses because of high cost and time consumption. The presence of spaces or gaps in the particles formed via freeze drying leads to the loss of essential oils, which makes this technique vulnerable and least preferred by industries [133]. In addition to the nanoencapsulation process, artificial intelligence and machine learning are important parameters to be considered for the efficient preparation of different nanoformulations with their stability and delivery strategies. Most importantly, the encapsulation of EOs involving a variety of techniques influences the physico-chemical properties of EOs like volatility, stability, sensory characteristics, and delivery statistics for improved antimicrobial properties, thus affirming their practical application in food and agricultural industries [134]. 

## 5. Application of Artificial Intelligence (AI) and Machine Learning (ML) in the Mathematical Modeling of Nanoemulsions

Artificial intelligence (AI) and machine learning (ML) are a hot topic that draws the attention of every reader. These two key tools are trending and finding a wide range of applications in the fields of science, technology, and innovation. With the launch of ChatGPT in recent years, this subject has gained popularity and is now often discussed globally and is being implemented in a variety of sectors, including academia. Artificial intelligence is basically the ability of any machine to think on the basis of mathematical algorithms or logic. This technology enables researchers to develop hypotheses, plan experiments, gather and analyze massive amounts of data, interpret results, and come to conclusions that otherwise might not have been possible or would be tiresome and time-consuming using only conventional scientific methods [135]. Hence, the integration of mathematical modeling with AI tools will enhance the understanding of nanoemulsion formulation, preparation strategies, and stability parameters, and it will provide insight into the release kinetics of essential oils or their bioactive components from nanoemulsions for improving their efficacy and efficiency in food systems. 

Researchers are investigating the complexities of machine learning approaches such as artificial neural networks (ANNs), genetic algorithms (GA), support vector machines (SVM), random forest, K-mean clustering, and decision tress to determine how they might be useful in designing improved food-delivery systems [136,137]. They are focusing on developing a ready-made machine-learning approach which can optimize all the parameters for nanoemulsion formulation. Artificial neural networks designed according to the structure and functionality of the human brain can serve this purpose. They contain neurons just like the human brain. ANNs can be primed with all the mathematical algorithms and can be trained to detect patterns in data, which can be utilized for prediction and decision-making. The power of ANNs has been exploited in the formulation of nanoemulsion and its optimization to predict the size of droplets in nanoemulsions, their stability, and various other parameters of the final emulsion depending upon the input variables such as type of essential oil (or bioactive compound), type of surfactant/co-surfactant used, and processing conditions [137,138]. A research study was conducted to optimize and anticipate the output of *Curcuma longa* L. essential oil in various agro-climatic zones through the use of ANNs. The data on essential oils from 131 germplasms of turmeric were collected from eight different agro-climatic zones of Odisha along with the soil conditions and environmental parameters, and an ANN-based prediction model was developed. The ANN model was trained and tested for eleven parameters for each sample. When the R^2^ value = 0.88, the results indicated that multilayer-feedback neural networks containing 12 nodes were the most appropriate and logical model to be utilized. This ANN-based prediction model can be explicitly utilized to estimate the yield of essential oil at a new location and to optimize oil yield at a particular location just by changing the input variables in the prediction model [139]. Another study was conducted to optimize clove essential oil nanoformulation and sonication conditions using RSM (response surface methodology), ANN, and ANFIS (adaptive neuro-fuzzy interference system). Four neurons were utilized for input variables in the prediction models, namely, surfactant concentration and type, sonication time, and temperature, and three neurons for output variables were utilized predicting accurately the mean size of droplet in nanoemulsion, PDI (polydispersity index), and dynamic viscosity [140].

An optimization technique called the genetic algorithm (GA) is based on Darwin’s theory of natural selection, which plays a vital role in evolutionary genetics [141]. It is a search algorithm that potentially utilizes the data of the existing population to predict the fitness of the futuristic population by applying selection pressure (i.e., survival of the fittest). By applying genetic operators repeatedly to the people already existing in the population, new generations can be reproduced. The major variables of GA are selection, chromosome representation, crossover, mutation, and fitness [142]. This concept of GA can be widely utilized in optimizing nanoemulsion formulations. It can be designed as follows:

➢ The input variables will be the components that can be varied in order to fabricate the nanoemulsion, such as type of essential oils, type of surfactant/co-surfactant used, concentration, sonication time, temperature, homogenization time, and speed, while the criteria for optimization should include variables like size of the droplet, stability parameters, loading capacity, encapsulation efficiency, polydispersity index, etc.
➢ The existing population will be a data set generated on the basis of pre-existing formulations available that would indicate the beginning of the optimization process.
➢ The fitness of any formulation will be evaluated by applying selection pressure (this selection pressure corresponds to measurements theoretically or through experimentations). The fitness score for any formulation will be an indicator of how well it fits the optimization criteria.
➢ The formulation with a high fitness score would be selected from the population.
➢ The selected formulations would be allowed to reproduce using key variables of GA, mutation (in which parent formulation is altered by sudden change in one or more components), and crossovers (in which two formulations are combined to reproduce a new formulation).
➢ The success rate of new offspring (formulations) will again be evaluated using the fitness score, and this process shall continue until end point criteria are fulfilled (stop signal may be defined as a set of generations or a stable formulation) [137].

W/O nanoemulsion was fabricated by combining UC (ultrasonic cavitation) and ISD (isothermal dilution process). The optimization process was carried out using an integrated genetic algorithm approach in which aqueous medium concentration, surfactant concentration, power density, and sonication time were taken as input variables. The prediction model was able to accurately determine the mean droplet size and the dynamic viscosity with less than 2.2% errors [143]. 

Support vector machine (SVM) is a machine-learning tool used for both classification and regression. This tool attempts to determine the best hyperplane to segregate the data points into various classes or groups [144]. The hyperplane strives for the widest feasible gap between the closest points of various classes. Extreme cases are considered support vectors. The number of attributes decides the dimensions of the hyperplane [145]. A study was conducted by researchers using the SVM approach to classify synthetic emulsions. The purpose of the study was to analyze the impact of non-ionic surfactant and Laponite clay on the stable nature of synthetic emulsions. Variable fractions of surfactant and clay were combined, 15 different emulsions were synthesized, and their stabilities were analyzed using RSM (response surface methodology). SVM was employed to classify emulsions into three categories, namely, stable, moderately stable, and unstable, to avoid errors and misclassifications frequently found during traditional classifications [146]. Similarily, K-mean clustering, random forest, decision trees, and many more machine learning algorithms can be applied to optimize nanoformulations. Thus, it can be concluded that the era of AI and ML will be of great importance to researchers, providing them with a platform to explore additional formulations, speed up the optimization process, and hasten the pace of discovery by minimizing the cost and time involved in generating new formulations and deep understanding of machine learning tools may result in technological advancements with better and refined use of nanoemulsions in various sectors, including nano-foods. The modeling of different encapsulating components along with controlled delivery has been supervised by artificial intelligence and machine learning, demonstrating the superiority of artificial neural networks with input–output relationships and practical implications [147]. 

## 6. Stability and Release Kinetics of Encapsulated Essential Oils

### 6.1. Stability

**Figure 3:**
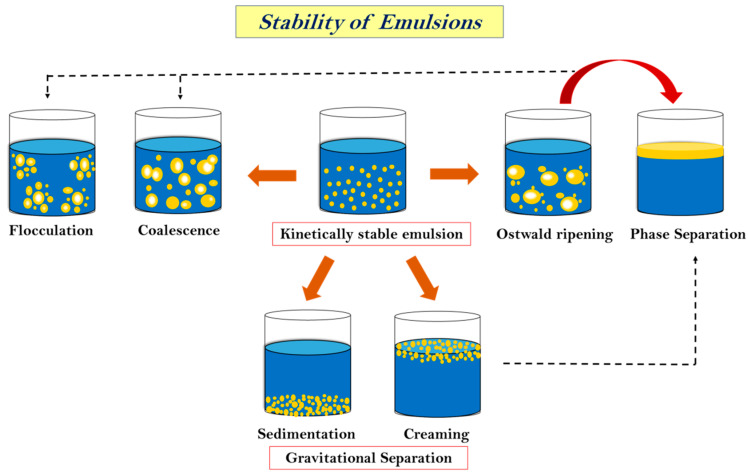
Stability of essential oil nanoemulsion.

The stability of an emulsion is a very critical aspect when we talk about its applications and storage. The capacity of emulsions to withstand alterations in the physicochemical attributes over time is known as stability. It also controls how long food emulsions last and how they are processed. Physical stability is the capacity to withstand changes in the spatial distribution of ingredients over time, whereas chemical stability is the capacity to withstand changes in the chemical structures over time [148]. The long-term stability of MEs and NEs is influenced by their distinct free energy levels [47]. MEs, which are thermodynamically stable under certain circumstances, will continue to be kinetically stable as long as the initial circumstances (like storage temperature and system composition) do not change [149]. The level of the energy barriers between the colloidal system and the separated phases will determine the long-term stability of NEs, which are thermodynamically unstable systems. These barriers will break down more quickly the lower they are [150]. High energy is achieved by avoiding contact and coalescence between droplets by repulsive interactions (hydrodynamic, steric, and electrostatic). Furthermore, as the frequency of interactions increases, the chances of instability of emulsion also increase. This is usually determined by the gravitational forces, Brownian motion, applied shearing, and prevailing temperature [82,151]. Gravitational separation (sedimentation), flocculation, coalescence, and Ostwald ripening are the primary instability phenomena that could lead to phase separation, as shown in Figure 3 [152]. Some of these phenomena are described below.

#### 6.1.1. Sedimentation or Creaming

Both of these processes are caused by gravitational effects and, hence, are categorized under gravitational separation. They are the most common forms of instability found in emulsions [153]. Creaming or sedimentation may occur depending on the relative densities of dispersed and continuous phases. If the density of the dispersed phase is less than the density of the continuous phase, droplets rise and are separated from the continuous phase, generating a distinct layer on the top of the continuous phase [152]. This is referred to as creaming. Sedimentation, on the other hand, occurs when the density of the scattered phase increases, causing the droplet to fall and create a layer at the bottom of the continuous phase [154]. Creaming velocity is used to express the rate of creaming, which may be estimated using Stoke’s law:

$$v=\frac{2gr^{2}\left(\rho_{w}-\rho_{0\;}\right)}{9\eta_{w}},$$

where *v* is the creaming velocity of the droplet, *g* is the gravitational acceleration, *ρ_w_* and *ρ*_0_ are the densities of the aqueous phase and oil phase, respectively, *r* is the droplet radius, and *η_w_* is the viscosity of the aqueous phase [56]. From the above equation, it is clear that gravitational separation can be reduced by decreasing the density differences between the two phases, decreasing droplet size, and increasing the viscosity of the continuous phase. Emulsifier coating provides a significant amount to the overall volume of the droplets, causing the density of the oil phase to alter. In that instance, the entire particle radius becomes:

$$r_{f}=r_{i}+\delta ,$$

where *r_f_* is the final droplet radius after coating, *r_i_* is the initial radius of the droplet, and *δ* is the thickness of emulsion coating on the droplet. The total density of a particle made up of an oil droplet encased in an emulsifier layer is computed as follows:

$$\rho_{total}={ɸ}_{shell}\rho_{shell}+\left(1-{ɸ}_{shell}\right)\rho_{core},$$

where *ρ_total_* is the total density of the particle, ɸ*_shell_* is the volume fraction of emulsion coating, and *ρ_shell_* and *ρ_core_* are the density of shell material used for coating and density of core material, respectively [152,155]. Typically, the density of the core material is lower than that of the shell material. Therefore, the surfactant coating increases the radius of the droplet, which leads to an enhancement in the droplet density.

#### 6.1.2. Flocculation and Coalescence

Flocculation is the separation of colloids or droplets into aggregates or flocs, either naturally or as a result of the addition of a reactive agent. When continuous-phase film ruptures, two droplets combine to form a larger droplet, which results in coalescence [150,156]. Depending on the intermolecular interactions between two droplets that come into contact, there may be flocculation or coalescence. If the intermolecular repulsive interactions are strong enough to maintain the droplet’s separation at a modest equilibrium distance, two droplets flocculate [157]. On the other hand, if intermolecular forces are exceedingly strong, the interfacial membrane is ruptured, causing two droplets to coalesce and form a larger droplet. The sum of four forces, namely, van der Waals attraction (*W_v_*), electrostatic repulsion (*W_e_*), steric hindrance (*W_s_*), and the attractive force due to hydrophobic interaction (*W_h_*), can be used to express the total energy of interactions (*G*) when two droplets with an interfacial membrane thickness of *δ* approach to a distance h [152].

$$G\left(h\right)=W_{v\;}\left(h\right)+W_{e\;}\left(h\right)+W_{s\;}\left(h\right)+W_{h\;}\left(h\right).$$

#### 6.1.3. Ostwald Ripening

The primary mechanism responsible for the instability of nanoemulsions is Ostwald ripening. This phenomenon involves the diffusion of the oil phase from small to larger droplets. As a result, the size of larger droplets increases with time at the expense of small droplets [158,159]. Laplace’s Law and the impact of pressure on dispersed phase solubility in the continuous phase is the main force responsible for Ostwald ripening. From Laplace’s Law, the pressure differential between the droplet’s interior and exterior can be computed as follows:

$$Pressure\;difference\;\left(\Delta P\right)=\frac{2\sigma }{radius},$$

where *σ* is the interfacial tension. From the equation, it is very clear that the radius of the droplet and the pressure difference are inversely related [148]. Hence, a decrease in droplet radius increases the interior pressure in the droplet, leading to increased solubility of the oil phase in the continuous phase. The Kelvin equation is used to predict the solubility (*S*) of the scattered phase in the continuous phase at the interface of droplet radius (*r*).

$$S\left(r\right)=S_{\alpha }e^{\frac{2\gamma V_{m}}{rRT}},$$

where *S_α_* is the dispersed phase solubility in the bulk of the continuous phase, *γ* is interfacial energy, *V_m_* is molar volume, *R* is the gas constant, and *T* is absolute temperature [160]. According to the Kelvin equation, the solubility of the dispersed phase increases as the radius of the droplet is reduced. Because of the difference in solubility of the two droplets, a concentration gradient is formed, which forces bulk diffusion to transfer from tiny droplets to larger ones. As a result of this bulk transfer, small droplets compress (reduce in size), and larger droplets grow, leading to the destabilization of the nanoemulsion system. The pace of Ostwald ripening is provided by the Lifshitz–Slyozov–Wagner (LSW) theory once a stable state is achieved.

$$r^{3}-r_{0}^{3}=\omega t=\frac{4}{9}\;\alpha \;S_{\alpha }Dt,$$

where *r* is the mean radius of the droplet at time (*t*), *r*_0_ is the radius of the droplet at time (*t* = 0), *D* is the diffusion coefficient of the dispersed phase, *ω* is the Ostwald ripening rate, and *α* = 2*γV_m_*/*RT* [161,162]. Ostwald ripening is the major cause of nanoemulsion destabilization. The major parameter determining this method of destabilization is the dispersed phase’s solubility in the continuous phase. The Ostwald ripening process in o/w nanoemulsions can thus be minimized or prevented by lowering the oil solubility in the aqueous phase of the solution. For example, long-chain triglycerides such as sunflower oils, corn, soy, fish oils, etc., are less prone to Ostwald ripening in o/w nanoemulsions because they have very low water solubility, while short-chain triglycerides like flavor oils and essential oils, being more soluble in water, are more prone to Ostwald ripening [163,164,165].

#### 6.1.4. Chemical and Biochemical Stability

Because nanoemulsions have such enormous surface areas, the effect of chemical breakdowns, such as lipid oxidation at the oil–water interface, is amplified [166]. Furthermore, nanoemulsions are sensitive to UV and visible light, promoting light-sensitive degradation of pigments or flavor compounds and hydrolysis of biopolymers [152]. To protect nanoemulsions against chemical instability, certain precautions need to be taken care of. Antioxidants or chelating compounds are usually added to nanoemulsions to improve the chemical stability of encapsulated labile components [148].

### 6.2. Controlled-Release Kinetic Models

#### 6.2.1. Burst Release

The uncontrolled release of bioactive compounds from different encapsulating matrices in a short duration of time constitutes burst release [167]. In case of burst release, the leakage of bioactives can range from 30 to 60% [168]. Generally, burst release follows a biphasic pattern, i.e., an initial ‘burst effect’ which is uncertain and rapid, followed by a sustained release profile. Usually, slow and constant release of bioactive compounds is favorable, while burst release patterns are usually disorganized in terms of economy as well as pharmacological perspective. Burst release models can be beneficial for localized drug penetration, for example, in the treatment of dermal infections, but sustained release is required for active drugs that can be lethal at high concentrations or in cases where prolonged drug release needs to be ensured because, in such cases, excessive burst release may lead to toxicity and can be life-threatening to raise numerous safety concerns [169]. A study conducted on limonene-containing amylose nanostructures by Ganje et al. [170] revealed that the amount of limonene released in the intestine decreased from 79% to 40%, which is roughly a fifty percent reduction, as the amylose nanocarrier size increased from 5.65 to 41.9 nm. The release occurred with a larger slope with higher consumption because decreasing the amylose levels improved the sensitivity of intestinal enzymes. By lengthening the sonication time, the mechanical stress accelerated the release of limonene from all samples. The initial burst release was caused by limonene entrapped between amylose helices. This occurred because larger particles contained more limonene molecules in between the amylose helices as compared to smaller ones. As a result, the former showed higher initial burst release.

#### 6.2.2. Sustained Release

Sustained release allows the distribution of bioactive molecules encapsulated in the biopolymeric matrix at a predetermined pace, resulting in the delivery of bioactives for an extended length of time [171]. A consistent pharmacological dosage within a therapeutic window is advantageous. Similarly, the design of flavor, substance-loaded delivery systems can better manage their release in foods. As a result, flavor compound migration at the oil–water interface as well as in the continuous phase, is crucial for attaining prolonged release [172]. Sustained release is also a promising method for reducing pharmacological side effects by minimizing fluctuations in the therapeutic concentration of bioactives in the body. The primary concept of a sustained drug delivery system is to optimize the biopharmaceutical, pharmacokinetic, and pharmacodynamics aspects of a drug so that its usefulness is maximized, adverse effects are avoided, and the target can be achieved [173]. Less variability in the drug level results in a more consistent pharmacological response, faster condition control, and minimum drug activity loss during continued usage [174]. This results in the increased bioavailability of any bioactive compound. For example, essential oils, due to their volatile nature and increased susceptibility towards light and oxygen, can be protected by encapsulating them in a polymeric matrix optimized for sustained release [175].

#### 6.2.3. Delayed Release

Delayed release is a method in which the active chemical is allowed to be released over a long period of time until its release is favored. In order to safeguard active drugs during gastric digestion and their release in the gastrointestinal system, delayed release is generally used [176]. A study conducted by Wang et al. revealed a noticeable delay in flavor release by up to three times longer when AMD, allyl methyl disulphide (a flavoring, lipophilic compound in garlic)-loaded lipid droplets were enclosed in biopolymer-based microgels [177]. The lipid oil droplet diameter in the emulsions (0.26 nm) was initially 1000-fold smaller than the microgel diameter (270 nm), which resulted in a substantially slower release rate. Additionally, physical interaction between the biopolymer network and the flavor molecules (AMD) results in binding, which leads to slow release and promotes a delayed release mechanism.

### 6.3. Models for Describing Release Kinetics of Essential Oils

The calculation and development of formulations, as well as assessing the release of essential oils or their various components in vitro and vivo experiments, can be carried out with the use of mathematical modeling [170]. It helps with the accurate fitting of experimental data to the planned models and the determination of key physical characteristics. Mathematical models are used to theoretically forecast the variables influencing controlled disassembly, such as the kind of bioactive components and encapsulants, as well as the network’s size and form [167]. The use of various theoretical, empirical, or semi-empirical equations allows for the evaluation of kinetic data on the release behavior of various bioactive components of essential oils from the encapsulating systems [178]. Crempei et al. [179] described the controlled release mechanism of rosemary essential oil from chitosan biopolymer by the Korsmeyer–Peppas model using the following equation:

$$C=k\times t^{n},$$

where *C* implies the amount of rosemary essential oil released at time *t*, and *k* is the release constant for the Peppas model. In this study, eight variants with variable concentrations of EO, chitosan, and Tween-80 were taken for investigation, which revealed that variant *V*_2_ (0.45% EO, 0.25% chitosan, and 1% Tween-80) depicts the best release behavior of bioactive compounds from chitosan matrix. This variant *V*_2_ follows Fick’s law of diffusion, and hence, this formulation can be utilized as a potent antibacterial in the food and cosmetic industries. A study was conducted on the release of lemongrass essential oil loaded in a sodium caseinate matrix. The amount of LMO released was fit into three different models, namely, Higuchi, Peppas, and Weibull, using the following equations:

(1) Q=kt12,

(2) $$Q=kt^{n},$$

(3) $$\ln(1-Q)=-a\times t^{b},$$

where *Q* = *M_t_*/*M*∞, M_t_ is the amount of LMO released at time *t*, and *M*∞ is the maximum amount considering all LMO to be released from microcapsules. The data for the release of LMO from the caseinate wall matrix demonstrated good agreement with the Weibull and Peppas model with a regression coefficient (*r*^2^) of 0.98. It was observed that by changing the constant b of the Weibull equation, which can be altered from 0.167 for microcapsules to 0.351 for films, the influence of the matrix on the release parameters of mathematical models can be easily detected. The LMO was also found to be a potent antibacterial agent, inhibiting the growth of *E. coli* and *Listeria monocytogenes* [180].

Various other equations and mathematical models describing the kinetic release behavior of essential oils or their bioactive components are presented in Table 1.

## 7. Advanced Application of Encapsulation Strategies in Food Preservation

Foods can be raw, processed, or prepared materials that are generally consumed by living organisms for their growth, development, health, contentment, pleasure, and social needs [187]. Since food is valuable to all living beings, it needs proper handling and preservation to fulfil the needs of every consumer. Hence, nanoencapsulation strategies have emerged as a novel and promising technology for the preservation of a variety of food commodities. Fresh vegetables and fruits are perishable because they are highly prone to decay due to the action of various microorganisms. Ripening of fresh fruits and vegetables can be efficiently delayed by edible coatings while retaining their nutritional properties. The addition of biologically active compounds, such as EOs, can further increase their efficiency [188]. However, various factors must be considered before they may be used in an in vivo food system, such as functional qualities, availability, cost, mechanical capabilities, resistance to water and germs, gas barrier properties, and sensory acceptability [189]. A recent study was conducted by Tabassum et al. [190] to prolong the shelf-life of freshly cut papaya. Nanoemulsion-based edible coating was formulated using alginate as wall material with different volumes of oregano essential oil (0.5, 1.0, and 2.0 mL). The results revealed that a combination of alginate with 1.0% (*v*/*v*) oregano essential oil was highly effective in extending the shelf-life of freshly cut papaya up to 16 days along with the preservation of its sensorial attributes. The color properties of the samples were predicted using machine learning tools such as ANN and SVR, which were found to be successfully fitted (R^2^ > 0.99) to the experimental data. Hence, the study proved the role of nanoemulsions as edible coatings and can be a promising solution to extend the shelf life of perishable fruits and vegetables. Arabpoor et al. [191] investigated the effect of *Eryngium campestris* essential oil (ECEO) encapsulated in chitosan nanoparticles (CHNPs) covering on cherry preservation at 4 °C; the CHNP-ECEO coating increased the pH, firmness, and antioxidant potential of cherries while decreasing the microbial load and reduction in weight. 

The rate of deterioration of fresh meat and fish food varies by species, but they are typically perishable foods because of their physico-chemical properties, serving as an excellent substrate for the growth of microbes. However, decomposition is frequently faster in fish since they are cold-blooded, and their metabolism is usually affected by alterations in temperature during storage conditions [10]. To enhance the shelf-life of meat and meat products, different strategies have been utilized for the inhibition of hazardous microorganisms [189]. Šojić et al. [192] identified an excellent combination that would enable the lowering of sodium nitrite levels in cooked pork sausages by integrating coriander essential oil (CEO). They optimized the combination by modeling ANNs. The ANN model comprised three input variables, namely, the concentration of sodium nitrite (NaNO_2_) (0, 50, and 100 mg/kg), the concentration of CEO (0.000, 0.075, 0.100, 0.125, and 0.150 µL/g), and storage time, and the output variables included pH, color (lightness (L*), redness (a*)), residual nitrite levels (RN), thiobarbituric acid reactive substances (TBARS), and microbial analysis in terms of total plate count (TPC). The results indicated that during 52 days of storage in a refrigerator, the combination of CEO (0.12 L/g) with a lower dosage of NaNO_2_ (60 mg/kg) was a highly effective formulation that slowed down the lipid peroxidation (TBARS approximately 0.12 mg MDA/kg) as well as microbial count (TPC approximately 2.50 Log CFU/g) and increased the redness of cooked pork sausages. Therefore, coriander essential oil could serve as a potential replacement for nitrite in the processing of meat and improve shelf-life due to its apparent antioxidant and antibacterial properties [192]. 

A study was conducted by da Silva et al. [193] to design biodegradable packaging by inclusion of EOs. The films were technologically sound for packaging purposes, opening up the possibility of utilizing the trash generated by the fish industry. The researchers used the k-nearest neighbors algorithm (KNN) as a reliable method for categorizing and choosing the best biodegradable packaging. The biodegradable packaging films were produced using gelatin (obtained from fish *Cynoscion acoupa)* infused with clove, palm, and oregano EOs. The film produced by the incorporation of clove essential oil in fish gelatin showed the most promising results using the KNN algorithm with high antioxidant potential, tensile strength, and elongation. The study also revealed that palm oil could be used as a potent alternative for designing biodegradable films due to its antibacterial properties, antioxidant activity, vast availability, and cost-effectiveness. Various other applications of encapsulated essential oils in food preservation are listed in Table 2.

## 8. Conclusions

The nanoencapsulation strategy has been demonstrated as an innovative technology to enhance the efficacy of essential oils and to avoid their limitations in food preservation. During nanoencapsulation, modeling of different nanoencapsulation parameters like surfactants, co-surfactants, the density of oil phase, and thickening agent facilitated the production of stable and efficacious nanoemulsions. Artificial intelligence and machine learning helped in mathematical modeling and algorithms for the prediction and decision-making procedure of different nanoformulation preparations. The article has also focused on technical principles of delivery of EOs from nanoemulsion with long-term efficacy in food systems. Moreover, the advanced application of different encapsulation strategies for the preservation of vegetables, fruits, muscle foods, and aqueous food systems has also been discussed for the practical utility of encapsulated essential oil in food and agricultural industries.

## Figures and Tables

**Table 1:** Mathematical models and equations for release kinetics of essential oils.

Kinetic Models	Equation	Specifications	Important Features	References
Zero-order	*Q_t_* = *k_A_* × *Q_A_*	*Q_t_* = cumulative percentage of released essential oil at time *t*; *k_A_* = rate constant; *Q_A_* = initial percentage of released essential oil; *t* = time	Release behavior of d-limonene from finger citron essential oil-loaded nanoemulsion was studied, which followed zero-order kinetics showing sustained release patterns.	[181]
First-order	ln(*A*) = ln(*A*_0_) − *kt*	*A* = percentage of essential oil released at time *t*; *A*_0_ = percentage of essential oil released at time 0; *k* = rate constant; *t* = time	Release of clove oil from ethyl cellulose microcapsules showed controlled release patterns along with better protection against volatility.	[182]
Second-order	*y*(*t*) = *k* [1 − (1 + *k*1(*t* − *d*))exp(−*k*1*t*)]	*y*(*t*) = percent creaming at time *t*; *k* = maximum value of creaming percentage reached during storage; *d* = time at which emulsion was completely stable; *k*1 = (1/day) constant of creaming process	Oregano essential oil was encapsulated in double emulsion (w/o/w), and the stability was analyzed. The creaming behavior during storage followed second-order kinetics from 1st day onwards, with maximum values between 15% and 35% and creaming rates between 3.16% and 7.3% per day.	[183]
Higuchi	*M_t_*/*M*∞ = *k_H_* × *t*^0.5^	*M_t_*/*M*∞ = percentage of essential oil released at time *t*; *k_H_* = Higuchi dissolution constant; *t* = time	The Higuchi model is based on Fick’s Law and makes use of pseudo-steady-state hypotheses to characterize the release kinetics of essential oils or any bioactive component from the porous matrix.	[37]
Kopcha	*M_t_* = *A* × *t*^0.5^ + *B* × *t*	*A* = rate constant for diffusion; *B* = rate constant for erosion; *t* = time	This equation is used to assess the diffusion or erosion-based release of bioactive components from delivery systems. The *A*/*B* ratio is employed to predict the predominant mechanism of essential oil release. *A*/*B* > 1 represents the diffusion mechanism; *A*/*B* < 1 represents the erosion mechanism; *A*/*B* = 1 represents that both diffusion and erosion will occur.	[184]
Avrami	ln (−ln *R*) = *n* ln *k* + *n* ln *t*	(−ln *R*) = retention factor; *n* = release parameter; *t* = time; *k* = rate constant	If the value of *n* = 1, it corresponds to first-order kinetics; *n* = 0.5, corresponds to diffusion mechanism; *n* ≤ 0.5 corresponds to the pseudo-Fickian diffusion mechanism.	[185]
Hixson-Crowell	(100 − *Q*)^1/3^ = − *kt* + *b*	*Q* = fractional release of essential oil in time *t*; *k* = rate constant; *b* = constant;	The Hixon–Crowell model suggests that the surface area of a spherical particle containing bioactive molecules is proportional to the cube root of its volume. This model is used to characterize dissolution release, assuming that the surface factors of spherical particles remain constant if dissolution is constant throughout the system.	[186]
Neibergull	(100 − *Q*)^1/2^ = −*kt* + *b*	*Q* = fractional release of bioactives in time *t*; *k* = rate constant; *b* = constant	Estimating the controlled release of bioactives from polylactic acid/chitosan nanoparticles.	[177]

**Table 2:** Applications of encapsulated essential oils in food preservation.

Essential Oil/Bioactive Component	Coating Material	Surfactant	Production Method	Energy	Food Product	Improvements during Preservation	References
Oregano essential oil (OEO) and resveratrol (RES)	Pectin	Tween-80	Magnetic stirring	Low energy	Pork loin	Encapsulation of OEO along with RES enhanced the preservation potential by effectively preventing protein and lipid peroxidation and maintaining the tenderness of meat with potential antimicrobial activity up to 20 days of storage.	[194]
*Ferulago angulata*	Chitosan	Tween-80	High-speed homogenization and ultrasonication	High energy	Rainbow trout fillets	A decrease in lipid peroxidation and total volatile nitrogenous compounds was observed. The encapsulated essential oil showed antibacterial activity, extending the shelf life of refrigerated rainbow trout fillets up to 16 days.	[195]
Grapefruit seed extract (GSE) or grapefruit seed oil (GEO)	Sodium alginate	Glycerol	High-speed homogenization	High energy	Table Grapes	Alginate coatings preserved the antioxidant activity of table grapes. The decay rate was successfully reduced. Effective in preventing weight loss and firmness with potent antifungal activity against *Penicillium digitatum*.	[196]
Lemongrass oil (LO)	Carnauba wax	Tween-80	Dynamic high-pressure processing	High energy	Plum	Reduction in weight loss, firmness, and lightness of plums. Maintained phenolic content in plums. Coatings were antibacterial in nature, preventing the growth of *E. coli* and *S. typhimurium.*	[36]
Lavender essential oil (LEO)	Gelatin	Tween-80	Magnetic stirring	Low-energy	Cherry tomatoes	Completely inhibited the growth of *S. aureus*, *L. monocytogenes*, and *E. coli*. Delayed deterioration of titratable acids and phenolic contents. Minimized the loss in weight and firmness and possessed high antioxidant activity.	[197]
Carvone	Chitosan	Tween-80	High-speed homogenization and ultrasonication	High energy	Bread	Carvone-loaded films completely inhibited *A. flavus* growth and aflatoxin B_1_ secretion. Improvement in gas composition and overall acceptable sensorial attributes in bread slices.	[198]
Cajuput essential oil (CjEO)	Chitosan	Tween-80	High-speed homogenization and ultrasonication	High energy	Mushroom (*Agaricus bisporus*)	CjEO-loaded chitosan nanoemulsion was able to preserve the quality of mushrooms for up to 7 days. Reduction in weight loss, firmness, and respiration rate while preserving the color and antioxidants in mushrooms.	[199]
*Foeniculum vulgare*	Basil gum and *Lepidium perfoliatum* gum	Polysorbate 80	Ultrasonication	High energy	*Oncorhynchus mykiss* Fish Fillets	The *Foeniculum vulgare* EO was found to be a potent antimicrobial agent against *Pseudomonas aeruginosa*, *E. coli*, and *Staphylococcus aureus* bacteria. The coating prevented the lipid peroxidation of fish fillets, and their antioxidant activity was enhanced.	[200]
*Valeriana officinalis* EO	Chitosan	Twen-80	Homogenization-based ionic gelation	High energy	*Citrus sinensis* fruits	The nanoemulsion coating improved the antifungal and anti-aflatoxigenic efficacy against toxigenic Aspergillus flavus along with the maintenance of fruit weight, titrable acidity, total soluble solids, and antioxidant enzymes in stored fruits.	[201]
Cinnamon essential oil	Pullulan	Twen-80	Homogenization followed by ultrasonic emulsification (ultrasound treatment)	High energy	Strawberry	Pullulan incorporated cinnamon essential oil and nanoemulsion enhanced the shelf life by reducing the senescence of fresh fruits at room temperature. The coating remarkably lowered the loss of fruit weight, total soluble solids, firmness, and titrable acids of fruits. Nanoemulsion-coated strawberries exhibited the strongest antimicrobial activity against bacteria and mold (2.544 and 1.958 log CFU/g, respectively).	[202]

## Data Availability

The data used to support the findings of this study can be made available by the corresponding author upon request.
